# Determination of the secondary structure of group II bulge loops using the fluorescent probe 2-aminopurine

**DOI:** 10.1261/rna.048306.114

**Published:** 2015-05

**Authors:** Abigael L. Dishler, Elizabeth L. McMichael, Martin J. Serra

**Affiliations:** Department of Chemistry, Allegheny College, Meadville, Pennsylvania 16335, USA

**Keywords:** bulge loops, secondary structure, thermodynamics

## Abstract

Eleven RNA hairpins containing 2-aminopurine (2-AP) in either base-paired or single nucleotide bulge loop positions were optically melted in 1 M NaCl; and, the thermodynamic parameters Δ*H*°, Δ*S*°, Δ*G*°_37_, and *T*_M_ for each hairpin were determined. Substitution of 2-AP for an A (adenosine) at a bulge position (where either the 2-AP or A is the bulge) in the stem of a hairpin, does not affect the stability of the hairpin. For group II bulge loops such as AA/U, where there is ambiguity as to which of the A residues is paired with the U, hairpins with 2-AP substituted for either the 5′ or 3′ position in the hairpin stem have similar stability. Fluorescent melts were performed to monitor the environment of the 2-AP. When the 2-AP was located distal to the hairpin loop on either the 5′ or 3′ side of the hairpin stem, the change in fluorescent intensity upon heating was indicative of an unpaired nucleotide. A database of phylogenetically determined RNA secondary structures was examined to explore the presence of naturally occurring bulge loops embedded within a hairpin stem. The distribution of bulge loops is discussed and related to the stability of hairpin structures.

## INTRODUCTION

Ribonucleic acid, RNA, is central to life processes. Among these are the control of gene expression (Hobert 2008), intron splicing (Schmelzer and Schweyen 1986; Doudna et al. 1989; Suzuki et al. 2006), protein synthesis and catalysis (Noller et al. 1992; Schuwirth et al. 2005), ligand binding (Hermann and Patel 2000a), and virus replication (Roy et al. 1990). Recent discoveries have highlighted the regulation of gene activity by miRNAs (Plasterk 2002; Matzke and Birchler 2005) and riboswitches (Winkler et al. 2004; Roth and Breaker 2009). Given its versatility in a wide range of biological functions, RNA has been recognized as much more than a passive intermediate between DNA and proteins.

The extraordinary functional capabilities of RNA can be attributed to its structural complexity. The most fundamental structural element in RNA is the duplex or stem, in which each nucleotide is base-paired with its complement. Other recurrent structural motifs include stem loops (hairpins), bulges, internal loops, pseudoknots, and multibranch loops. It should be noted that these motifs are likely to be crucial to the evolution of modern organism and studies have underscored their importance in a variety of biological events (Moore 1999; Leontis et al. 2006).

Bulges are regions of unpaired nucleotides situated along one strand of a duplex and are often found at protein binding sites (Wu and Uhlenbeck 1987; Lilley 1995). The most common bulge in nature consists of a single nucleotide. Both single and multiple nucleotide bulges in RNA are known to affect the stability of the backbone and the manner in which the RNA folds (Barthel and Zacharias 2002). To increase protein–RNA interactions, a bulge may alter the uniform helical arrangement of a duplex (Wu and Uhlenbeck 1987; Lilley 1995). This permits base pairs within the major groove to be more exposed, and thus easier for proteins to access by providing a greater surface area (Hermann and Patel 2000b). For example, the transactivator protein, Tat, increases the rate of HIV viral gene expression. Tat binds to a region of RNA known as the transactivation response element (TAR) that contains a 3-nt bulge. This bulge is necessary for Tat protein to recognize and bind TAR and for viral gene expression and replication to occur (Richter et al. 2002).

Single nucleotide bulge loops have been characterized by their structural characteristics into three classes, intercalated, extrahelical, and side-by-side (base triple) arrangements (Znosko et al. 2002). In addition to their structural diversity, bulge loops also have sequence characteristics that define the bulge position and possible ambiguity (Blose et al. 2007). Bulge loops have been divided into four groups based upon this sequence variability. Group I bulge loops have no sequence ambiguity and the identity of the bulge is known. For example, with 5′ C**A**U/3′ G A, the bold **A** is the bulged nucleotide. For groups II, III, and IV, the identity of the bulge and the base pairs adjacent to the bulge leads to ambiguity to the position and/or the identity of the bulge. For example, 5′C**AA**/3′G U, 5′ C**AG**/3′G U, and 5′**GAA**/3′U U, where the bold bases are the potential bulge, are typical group II, III, and IV bulge loop sequences, respectively.

The current work is a continuation of our studies on the stability and structure of bulge loops in the context of an RNA hairpin stem (Lim et al. 2012; Kent et al. 2014). A simple model has been proposed to predict the influence of bulge loops on the stability of hairpin formation that depends upon non-nearest-neighbor interactions (stability of the stem distal to the hairpin loop) (Kent et al. 2014). In-line structure probing has been used to determine the identity of the bulge in cases where there was ambiguity (group II or III bulge loops). For both group II and III bulge loops, the base farther from the hairpin loop was more susceptible to cleavage, indicative of the bulged nucleotide. In this study, the fluorescent properties of the base analog 2-AP provided a sensitive structural probe for the local environment of the base. The changes in fluorescent intensity upon denaturation of the hairpins containing group II bulge loops were consistent with the nucleotide farther from the hairpin loop being unpaired.

## MATERIALS AND METHODS

### RNA synthesis and purification

Most oligomers were synthesized on CPG solid supports (Applied Biosystems 392 DNA/RNA Synthesizer) utilizing phosphoramidites with the 2′ hydroxyl protected as the *tert*-butyl dimethylsilyl ether from Glen Research (Usman et al. 1987; Wincott et al. 1995). Oligomers underwent ammonia and fluoride deprotection, and crude sample was purified using preparative *tlc* (*n*-propanol:ammonium hydroxide:water, 55:35:10) and Sep-Pak C18 (Waters) chromatography. Some oligomers were ordered from Dharmacon and deprotection of the oligomers was carried out using the manufacturer's instructions. The oligomers were then purified as described above. Sample purity was determined through analytical TLC or HPLC (PRP-1 [Hamilton]), and was >95%.

### Melting curve and data analysis

Optical melting experiments were performed using a Beckman DU 640 Spectrophotometer and High Performance Temperature Controller at 260 or 280 nm. Absorbance changes for oligomers in 1 M NaCl melt buffer (1 M NaCl, 0.02 M cacodylic acid, 0.001 M EDTA, pH 7.0) were recorded as a function of temperature from 10°C to 90°C at a rate of 1°C/min as described previously (Serra et al. 1994). The experiment was repeated at 10 varying sample concentrations to give at least a 50-fold concentration range (10 μM–1 mM) for each sample. The thermodynamics were found to be independent of RNA concentration, indicative of a unimolecular hairpin transition. Absorbance versus temperature profiles were fit to a two-state model with sloping base lines using a nonlinear least squares program (McDowell and Turner 1996). Thermodynamic parameters for hairpin formation were obtained from the fits of the individual melting curves.

### Determination of the contribution of bulge loops to duplex thermodynamics

The free energy contribution of each bulged nucleotide, $ƛ\Delta G{{}^{\circ}}_{37(\text{bulge})},$ was calculated from the experimental data according to Equation 1, where $ƛ\Delta G{{}^{\circ}}_{37(\text{bulge hairpin})}$ is the experimentally determined value from the melts and $ƛ\Delta G{{}^{\circ}}_{37(\text{parent})}$ is the measured value for the parent strand.

(1) $$\Delta G{{}^{\circ}}_{37(\text{bulge})}=\Delta G{{}^{\circ}}_{37(\text{bulge hairpin})}-\Delta G{{}^{\circ}}_{37(\text{parent})}\;(1)$$

### Fluorescent melts

Fluorescent measurements were performed using a Shimadzu RF-501spectrofluorometer and a water bath for temperature control. Fluorescent melts were carried out at an excitation wavelength of 303 nm and an emission wavelength of 370 nm. The excitation and emission slits were 5 nm. Samples were heated from 0°C to 85°C at a rate of 1°C/min using the same buffer conditions as the optical melts. The fluorescence of each RNA hairpin was normalized to a 5 nt short strand of RNA that was analogous to the region of the RNA hairpin where the 2-AP was inserted.

### Phylogenetic analysis

The RNA Strand database of RNA secondary structures (Andronescu et al. 2008) containing 4666 structures of which 2898 contained bulge loops was analyzed. The database was searched for single nucleotide bulge loops. A total of 20,292 bulge loops was identified. The loops were characterized by bulge group type (I, II, or III) and location within a hairpin stem.

## RESULTS

The thermodynamics for hairpin formation for 21 RNA hairpins containing either A or 2-AP in either the 5′ or 3′ stem of the hairpins are listed in Table 1. The average deviations in thermodynamic parameter values for the hairpins with group I bulge loops or fully base-paired stems are 4.8, 13.3, and 0.3 for Δ*H*°, Δ*S*°, $\Delta G{{}^{\circ}}_{37},$ respectively. The average errors for the hairpins with group II bulge loops are larger (7.6, 24.2, and 0.4 for Δ*H*°, Δ*S*°, $\Delta G{{}^{\circ}}_{37},$ respectively) than for the fully base-paired or group I bulge loops perhaps indicative of some slight non-two-state melting (Kent et al. 2014).

**TABLE 1.**
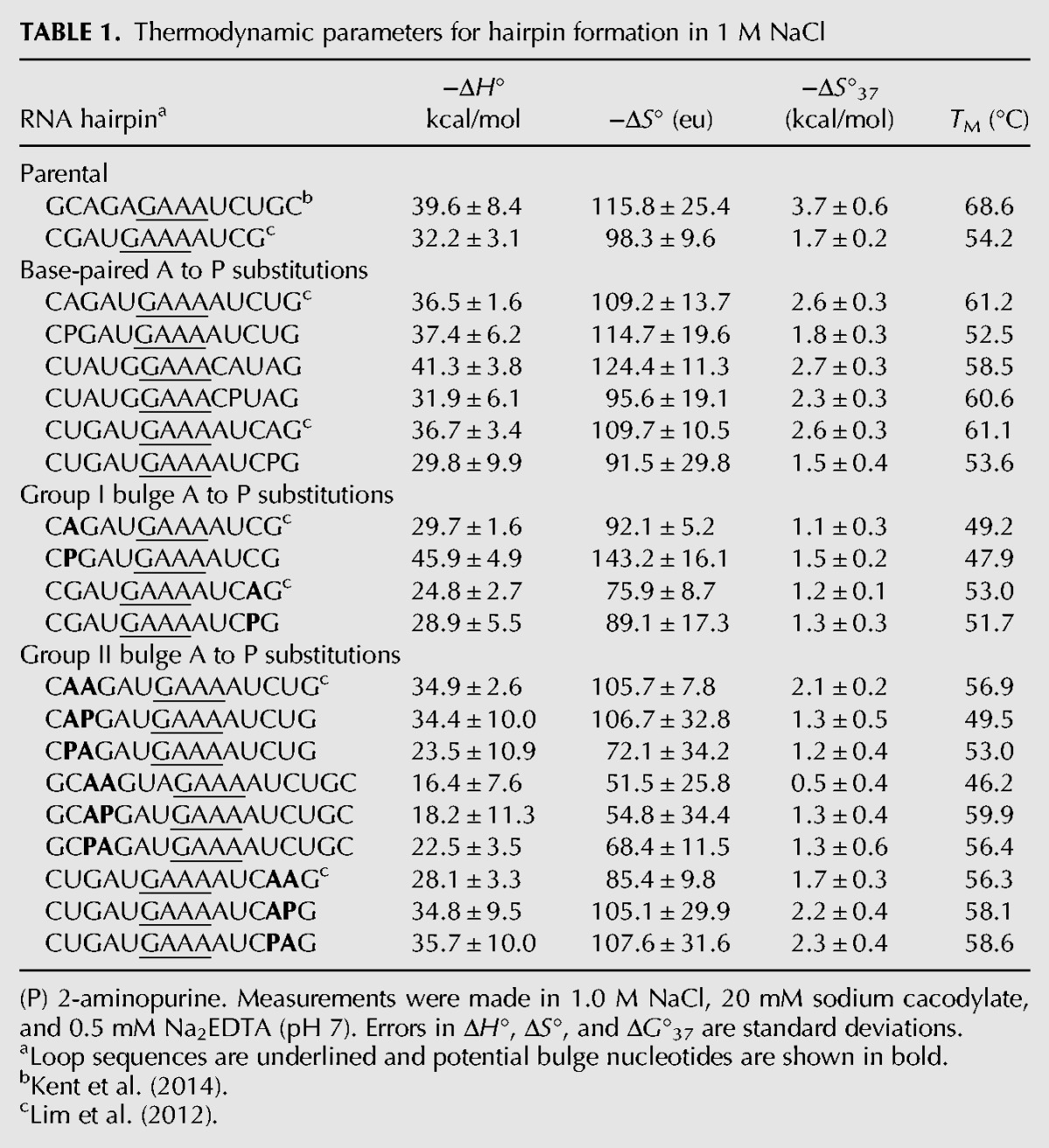
Thermodynamic parameters for hairpin formation in 1 M NaCl

### Influence of 2-AP on hairpin stability

Three of the fully base-paired parent hairpins (two on the 3′ side of the hairpin loop and one on the 5′ side) in Table 1 have 2-AP substituted for A. The average difference in stability for the three sets of hairpins is 0.8 kcal/mol with the 2-AP being less stable than the A hairpins. This difference is the same on average for the 3′ or 5′ substituted hairpins. The differences in stability between the A and 2-AP substituted hairpins are larger for the two hairpins where the substitution is near the terminus of the hairpin stem (1.0 kcal/mol) while substitution into the middle of the hairpin stem was only a 0.4 kcal/mol difference. The greater flexibility at the hairpin terminus may allow for larger differences in environment and therefore larger differences in stability of the 2-AP purine residue.

Two group I bulge loop hairpins (one on the 3′ side and one on 5′ side of the hairpin) have 2-AP substituted for A as the bulge in Table 1. In both cases, the differences in stability between the 2-AP and A bulged hairpins are not significant (0.25 kcal/mol on average).

Finally, six group II bulge loop hairpins (four on the 5′ side and two on the 3′ side of the hairpin loop) have 2-AP substituted for A as one of the possible bulged nucleotides. Substitution of 2-AP into the bulge loop for one of the A residues on the 5′ side of the hairpin stem leads to either an increase or decrease in stability by 0.8 kcal/mol. While 2-AP substitution on the 3′ side of the stem results in a slight increase in stability (0.6 kcal/mol on average). Placement of the 2-AP in the proximal or distal position of the group II bulge loop, relative to the hairpin loop, has no effect on the stability of the hairpin for either the 5′ or 3′ group II bulge loops. In all six examples, the nucleotides surrounding the bulge are the same suggesting the differences must be related to non-nearest-neighbor influences such as the placement of the bulge in the hairpin stem. More studies will need to be done to elucidate the influence of 2-AP on hairpin formation.

### Modulation of 2-AP fluorescence by thermal denaturation of RNA hairpins

The fluorescence of 2-AP is sensitive to its local environment (Menger et al. 2000; Ballin et al. 2007; Sarkar et al. 2010; Rau et al. 2012). The change in fluorescence with temperature has been used to examine the change in local structure as a molecule denatures. Typically, when a base-paired 2-AP base denatures, the fluorescence intensity of the base increases due to the decrease in stacking of the base with its neighbors; conversely, an unpaired 2-AP base decreases its fluorescence as it denatures due to increased interactions with its neighbors in the single-stranded form (Menger et al. 2000; Jiao et al. 2002; Hardman and Thompson 2006; Ballin et al. 2007; Sarkar et al. 2009, 2010; Rau et al. 2012; ). The major goal of this study was to use the change in 2-AP fluorescence to investigate the structural ambiguity of the group II single nucleotide bulge loops.

**FIGURE 1.**
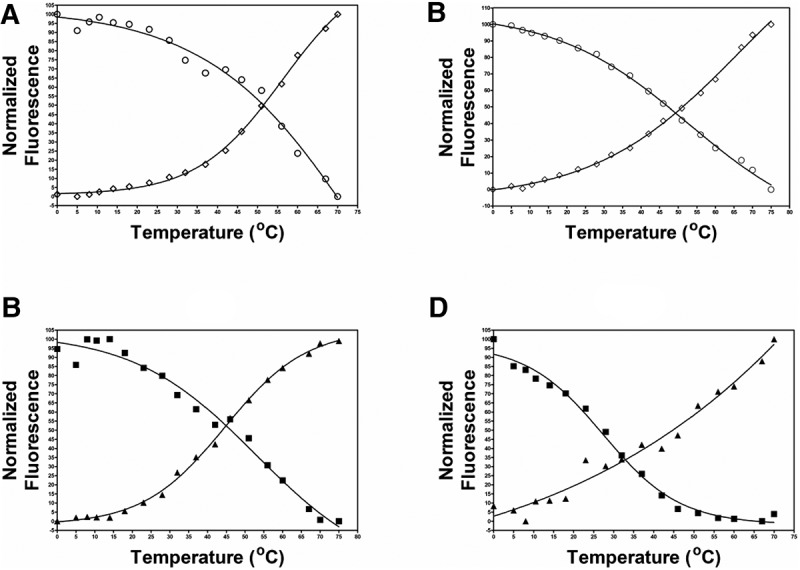
Thermal denaturation analysis of 2-AP substituted RNA hairpins. (*A*) (○) C**P**GAUGAAAAUCG, (◊) CPGAUGAAAAUCUG. (*B*) (○) CGAUGAAAAUC**P**G, (◊) CUGAUGAAAAUCPG. (*C*) (▪) C**PA**GAUGAAAAUCUG, (▴) C**AP**GAUGAAAAUCUG. (*D*) (▪) CUGAUGAAAAUC**AP**G, (▴) CUGAUGAAAAUC**PA**G. Fluorescent intensity values were normalized to emission from pentamer control RNA sequences at each measured temperature.

In addition to local environment, the fluorescence of 2-AP is strongly influenced by temperature (Menger et al. 2000; Ballin et al. 2007; Sarkar et al. 2010; Rau et al. 2012). Therefore, to accurately use 2-AP fluorescence to monitor the structural changes, fluorescent measurements for the 2-AP labeled RNA hairpins were corrected for the fluorescence of a five nucleotide short strand of RNA that was analogous to the region of the RNA hairpin where the 2-AP was inserted (Ballin et al. 2007). Figure 1 displays the results of the fluorescence melts. Panel A (5′ side of the hairpin loop) and B (3′ side of the hairpin loop) show the results for bulged and paired 2-AP control hairpins. In both cases, the change in fluorescence shows the expected behavior. As the paired 2-AP stem undergoes melting, the 2-AP interacts less strongly with its neighbors and the fluorescent intensity of the hairpin increases. The bulged 2-AP shows a decrease in fluorescent intensity upon melting, since the bulge interacts more strongly with its neighbor in the single-stranded form.

Figure 1C displays the results for two group II bulge loop hairpins labeled with 2-AP in either the distal or proximal bulge position on the 5′ side of the hairpin loop. The change in fluorescence for the two hairpins is consistent with the bulged nucleotide being the one farther from the hairpin loop. Figure 1D displays a similar set of hairpins but with the group II bulge loop on the 3′ side of the hairpin loop. Again, the fluorescent change is consistent with the bulged nucleotide being the one farther from the hairpin loop.

The results of fluorescent melts of the other 2-AP labeled hairpins listed in Table 1 are summarized in Table 2. The changes in fluorescent intensity are consistent with the results shown in Figure 1; paired 2-AP residues display an increase in fluorescence intensity upon melting and bulged 2-AP residues display a decrease in fluorescence intensity upon melting. The change in fluorescent intensity is greater when the 2-AP is base-paired (average change 150% increase) than for the group I 2-AP bulged (average change 27% decrease). This suggests that the environment of the 2-AP is undergoing a greater change in going from base-paired to single stranded than from bulged to single stranded. This is not unreasonable as the bulged base is probably stacked, although perhaps not optimally, in the duplex (see Discussion). The change in fluorescent intensity upon melting of the group II bulge loops show the expected increase or decrease consistent with the bulged nucleotide being the one further from the hairpin loop. The extent of change for the group II “paired” 2-AP is smaller (on average 35%) than that seen for base-paired 2-AP. The difference in the extent of change in fluorescent intensity may be indicative of some structural heterogeneity, as noted in the larger than usual measured errors in the thermodynamic melts for the group II bulge loop hairpins.

**TABLE 2.**
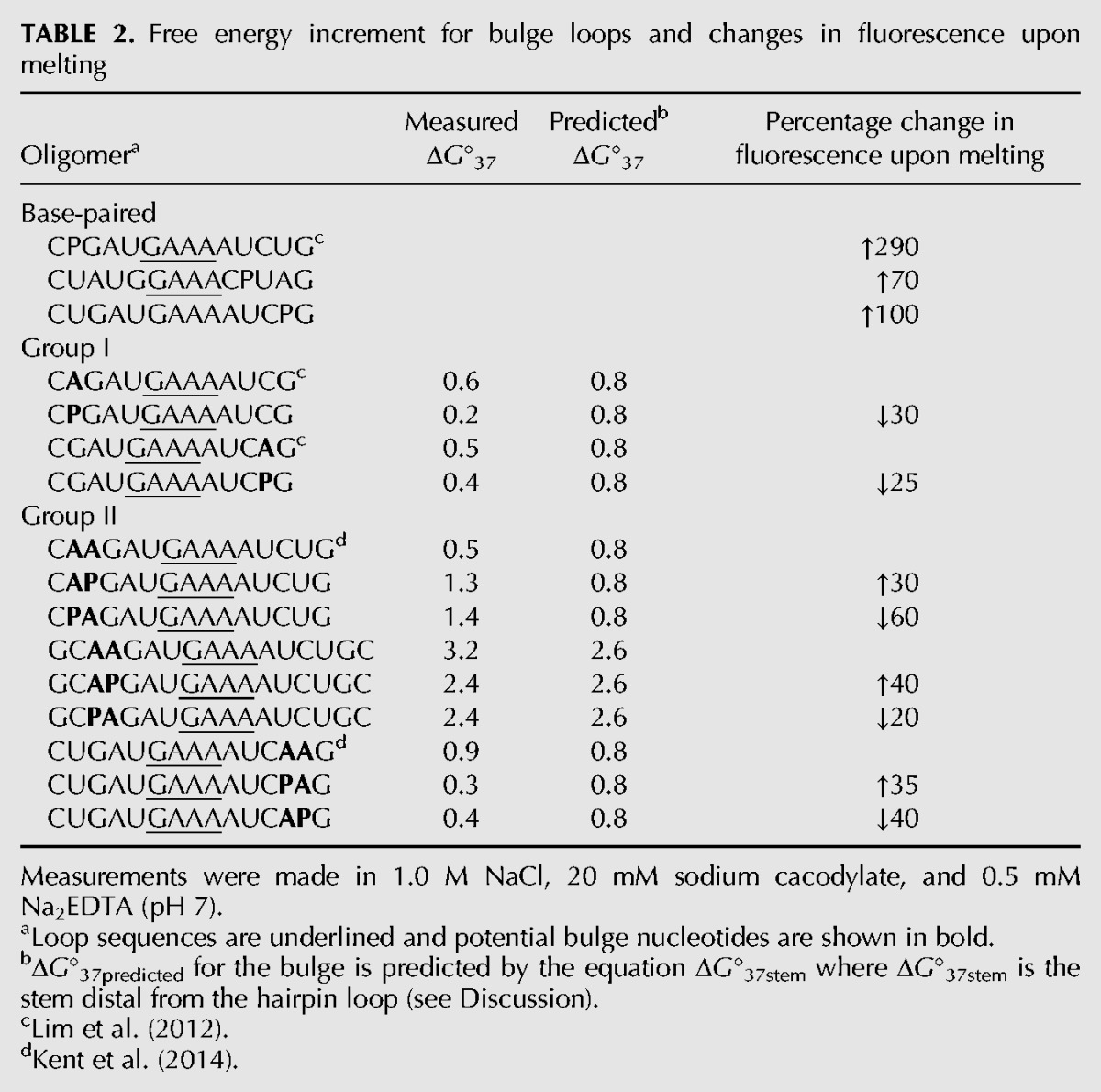
Free energy increment for bulge loops and changes in fluorescence upon melting

The substitution of 2-AP in either the proximal (base-paired, stacked) or distal (bulged, not stacked) position of the group II bulges leads to nearly the same change in thermodynamic stability of the hairpins (Table 2); therefore, it is not possible to relate the structure of the 2-AP to changes in thermodynamic stability for the group II bulge loops.

### Phylogenic analysis of single nucleotide bulge loops

To further characterize bulge loops, a database of RNA secondary structures was examined. The database contained 20,292 single nucleotide bulge loops. As previously observed (Gutell et al. 2000; Blose et al. 2007), the most prevalent bulge loop is adenosine, it occurred 9161 times in the database representing 45.3% of all of the single nucleotide bulge loops. The next most prevalent bulge loop was U occurring 4806 times (23.8%), followed by G occurring 4074 times (20.1%) and finally C, which was the least prevalent occurring only 2186 times (10.8%).

The database contained 6994 group II single nucleotide bulge loops representing 34.5% of all single nucleotide bulge loops. They are almost evenly divided between structures where the 5′ nucleotide is paired (3754) and the 3′ nucleotide is paired (3240). The AA group II bulge is the most prevalent but by a smaller percentage (25.4%) than was observed for the A bulges in total (Table 3).

**TABLE 3.**
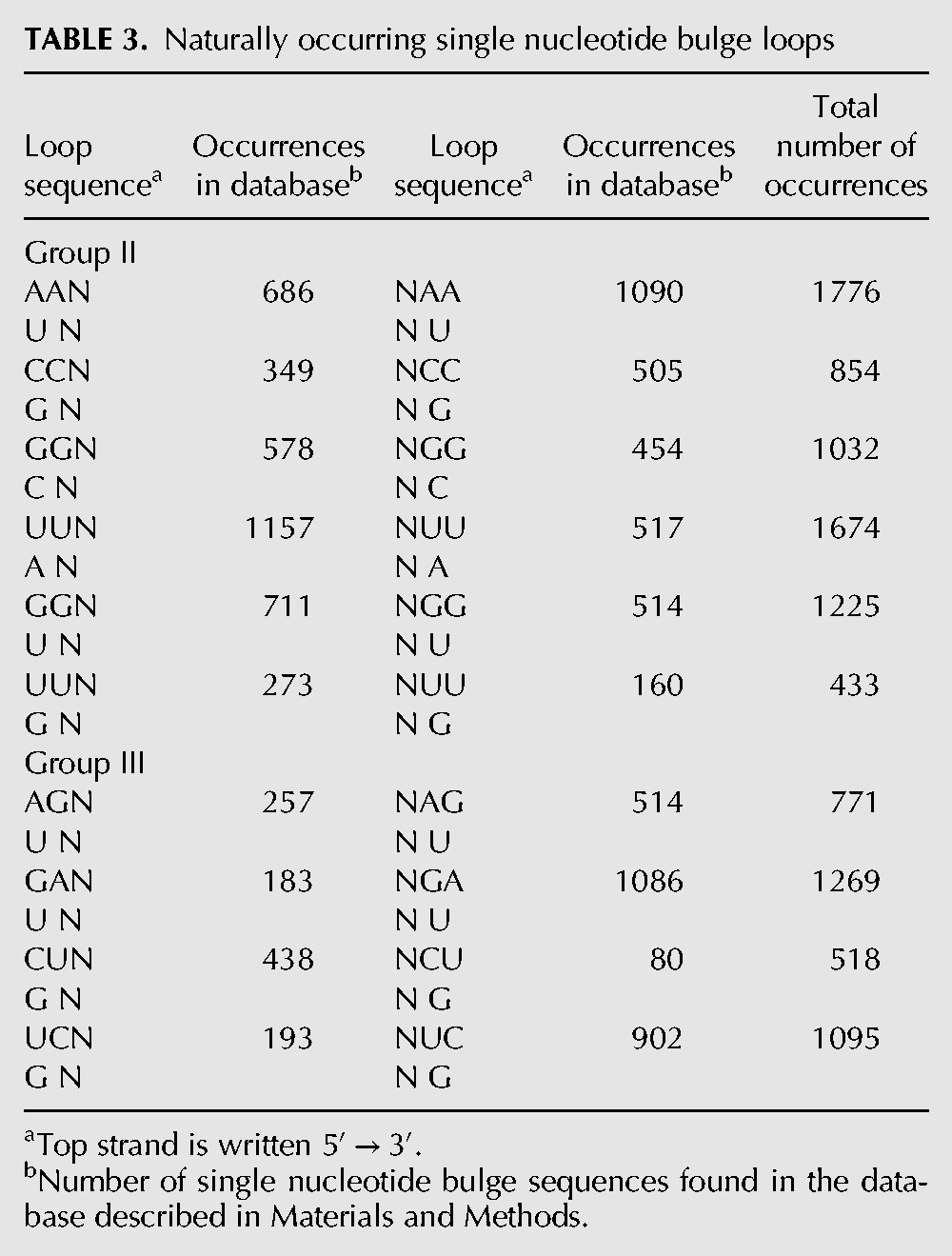
Naturally occurring single nucleotide bulge loops

In the case of Group III single nucleotide bulge loop, the identity of the bulge is ambiguous and the bulge may have either a Watson–Crick or wobble base pair. In the database there are 3653 group III bulge loops. Over 2/3 of the group III bulge loops have the nucleotide on the 3′ side paired. Nearly 75% (2683) of the group III bulge loops form a Watson–Crick rather than wobble base pair (Table 3).

Since this investigation was examining the influence of a bulge loop in the context of a hairpin stem, we next examined the natural occurrence of bulge loops in hairpin stems in the database. Table 4 displays the results of this analysis. The database was examined for distance (1–8 bp) of the bulge loop from hairpin loops of various sizes (3–10 nt). Nearly the same number of bulge loops is found on the 5′ (1756) or 3′ side (1799) of the hairpin stem. The total number of bulge loops found in hairpin stems (3555) is only 17.5% of the total number of bulge loops in the database. This is somewhat surprising as the database is dominated by rRNA and tRNA structures with >70% of the nucleotides present in hairpin structures.

**TABLE 4.**
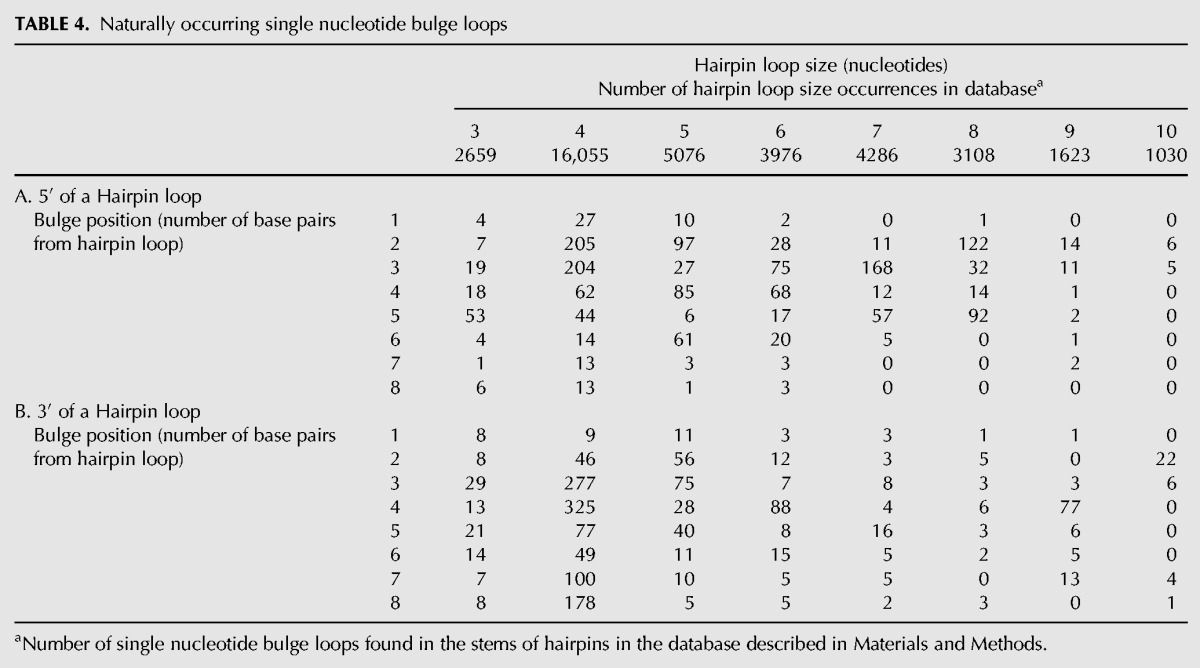
Naturally occurring single nucleotide bulge loops

Group II bulge loops are very rarely embedded in hairpin stems (for hairpin loops up to 7 nt long and bulges up to 6 bp from the hairpin loop). Only 419 examples are found in the database, they are almost evenly divided as to whether the bulge is on the 5′ (195) or 3′ (224) side of the hairpin loop. There is also no discernible preference for whether the nucleotide proximal (213) or distal (206) relative to the hairpin loop is the bulged nucleotide (data not shown).

## DISCUSSION

### Influence of bulge loops on hairpin stability

2-AP can interact with uridine with Watson–Crick geometry and forms two hydrogen bonds similar to adenosine. Substitution of 2-AP for A has been shown previously to have a small influence on the stability of RNA (Zagórowska and Adamiak 1996; Jiao et al. 2002; Liu et al. 2008; Sarkar et al. 2010). It was important to determine if the 2-AP/U and A/U base pair were similar in stability in the context of a hairpin stem. For the three hairpins with fully base-paired stem, the 2-AP hairpin was 0.8 kcal/mol less stable than the corresponding A hairpin (Table 1). These results are similar to the effect of 2-AP on DNA duplex stability where substitution of 2-AP for A decreases the stability of DNA duplexes (Law et al. 1996; Sowers et al. 2000).

Incorporation of a 2-AP:U base pair at the terminal position of a RNA duplex increases the stability of the duplex by nearly the same amount as the addition of a terminal A/U base pair (Liu et al. 2008). The differential effect of 2-AP substitution at a terminal position may be due to the increased dynamics of the terminal base pair. The range of effects we observed for the substitution of 2-AP for A into base-paired positions in hairpin stems presented in Table 1 (0.4–1.1 kcal/mol) suggests that there are nearest-neighbor and non-nearest-neighbor influences which need to be investigated more thoroughly.

Table 2 displays the influence of insertion of an A or 2-AP bulge loop into the hairpin stem. Substitution of 2-AP for A at Group I bulged positions does not significantly influence the stability of the RNA hairpin relative to the bulged A hairpins. This is not surprising, as we have previously shown that the identity of the bulge does not influence RNA duplex stability (Blose et al. 2007; Lim et al. 2012). The insertion of the group I bulge (A or 2-AP) decreases the stability of the hairpin by on average 0.4 kcal/mol. We previously developed a model to predict the influence of single nucleotide bulge loops on the stability of duplex and hairpin formation (Kent et al. 2014). That relationship is given in the equation below:

(2) $$\Delta G{{}^{\circ}}_{37\;\text{bulge loop}}=-0.51\Delta G{{}^{\circ}}_{37\;\text{stem}}+0.85,$$

where $\Delta G{{}^{\circ}}_{37\text{stem}}$ is the less stable stem for group I bulge loops and the second least stable stem for group II and group III bulge loops. For hairpins with embedded bulge loops, the $\Delta G{{}^{\circ}}_{37\text{stem}}$ is calculated for the distal stem (Lim et al. 2012; Kent et al. 2014). Note that with this model, the bulged nucleotide does not disrupt the nearest-neighbor interactions of the base pairs neighboring the bulge loop. For example, the hairpin C**A**GAUGAAAAUCG, where **A** is the bulged nucleotide and the underlined bases are the hairpin loop, the distal stem would be a C/G base pair. Since the C/G base pair has no nearest neighbor, its nearest neighbor stability is defined as 0, therefore using 0 in Equation 2, the predicted value for the influence of the bulge loop is 0.8 kcal/mol.

Using the model for determining the influence of single nucleotide bulge loop on duplex or hairpin stability (Equation 1), the bulge does not disrupt its nearest-neighbor interactions. Therefore, in this example, the stability of parental hairpin, CGAUGAAAAUCG, is just the measured value determined from the optical melt. There is good agreement between the measured and predicted values for the effect of group I bulge loops (A or 2-AP) on the stability of the RNA hairpins (average difference of 0.4 kcal/mol). This analysis suggests that the terminal CG base pair does form and in fact interacts with its nearest neighbor in a similar manner whether or not the bulge is present. Our previous in-line probing results have not detected any enhanced cleavage of terminal base pairs, again indicative of the fact that the terminal base pair does not undergo any enhanced fraying as a result of the bulge loop (Lim et al. 2012; Kent et al. 2014).

Table 2 also presents the influence of the 5′ AA, 5′ A 2-AP, and 5′ 2-AP A group II bulge loops on the stability of hairpin formation. Due to the ambiguity of the group II bulge loops, the identity of the hairpin stems is uncertain. However, both in-line probing (Lim et al. 2012) and the current fluorescent measurements (Fig. 1) suggest that the nucleotide farther from the hairpin loop is the bulged nucleotide. If the nucleotide farther from the hairpin loop is considered to be the bulged nucleotide, then Equation 2 can be used to predict the influence of the bulge on hairpin stability. The predicted values are listed in Table 2. There is good agreement between the measured and predicted values for the effect of group II bulge loops on the stability of RNA hairpins (average difference 0.4 kcal/mol). Therefore, the model developed for predicting the influence of bulge loops on RNA stability (Equation 2), which is nucleotide independent, also works well to predict the influence of synthetic bulged nucleotides (2-aminopurine).

### Identification of bulge nucleotide in group II bulge loops

The main goal of this study was to resolve the structural ambiguity present in the group II bulge loops. We previously have used in-line probing to identify the bulged nucleotide (McCann et al. 2011). In the context of a GNRA tetraloop hairpin, the nucleotide farther from the hairpin loop was identified as the bulged nucleotide. The results from this study, using fluorescence to monitor the environmental changes in 2-AP as the GNRA RNA hairpin unfolds, confirms the results obtained with the in-line measurement. For group II bulge loops on either the 5′ or 3′ side of the hairpin stem, the change in fluorescent intensity as the hairpin unfolds is consistent with the nucleotide farther from the hairpin being the bulged nucleotide (Fig. 1).

The group II bulge nearest-neighbor sequences in this study were chosen such that whichever nucleotide was bulged, the stability of its nearest-neighbor interactions would have similar stability. For example, for the hairpin C**AA**GAUGAAAAUCUG, if the first **A** was the bulge nucleotide its nearest-neighbor sequence would be 5′CA/3′GU which has a nearest-neighbor stability value of 2.11 kcal/mol; while if the second **A** was the bulge nucleotide its nearest-neighbor sequence would be 5′AG/3′UC which has a nearest-neighbor stability value of 2.08 kcal/mol. That the distal A residue is consistently found to be the bulged nucleotide is therefore not related to stability of the potential nearest-neighbor interactions for the examples we have studied (e.g., stable GNRA [GAAA] tetra loop and adenosine bulge loop). We are currently investigating the structural and sequence parameters that influence the positioning of the bulge loop.

### Naturally occurring bulge loops

We previously examined the bulge loop distribution of a much smaller database (Blose et al. 2007; McCann et al. 2011). The current database of known sequences is almost 10 times larger and also contains a more diverse group of RNAs than used in our previous analysis. This should be more representative of the distribution of bulge loops in nature. As previously observed, adenosine is by far the most prevalent bulged nucleotide (Gutell et al. 2000; Blose et al. 2007). There are 6994 group II bulge loops in the current data base and several interesting observations can be made (Table 3). First, the number of group II bulges that form a base pair using the 5′ nucleotide is almost identical to the number using the 3′ nucleotide to form the base pair. Second, GG is the most prevalent group II sequence; 2257 are GG, while only 1776 are AA bulges. Note that GG may have either a C or a U nucleotide on the opposite strand; in fact, the GU wobble motif is the more prevalent. While it is not clear why the GG group II bulge is so prevalent, it may be related to the ability of G to form specific interactions in the context of an ambiguous position (see below). Of the 2683 group III bulge loops identified in Table 3, three-fourths of the bulges form base pairs using the potential Watson–Crick base pair. This preference is even more pronounced for the bulge loops with potential to form the more stable GC base pairs (83% of the loops form the Watson–Crick rather than wobble base pair).

Examining bulge loops in the context of a hairpin stem, the first and most striking observation is the dearth of bulge loops in hairpin stems found in the database (Table 4). Only 17.5% of the bulges in the database are present in hairpin stem motifs. It is not clear whether this is a function of the database, which is largely weighted toward tRNA and rRNA sequences or other factors. Of the bulge loops present in the hairpin stems, the bulges are equally distributed between the 5′ and 3′ side of the hairpin loop. This is congruent with the thermodynamics of bulge loops, as incorporation of a bulge loop into a hairpin stem has similar influences on the thermodynamics of hairpin formation whether the bulge is inserted into the 5′ or 3′ side of the hairpin loop (McCann et al. 2011; Lim et al. 2012; current study).

Tetraloop hairpins have the highest number (1643) of bulge loop embedded in their stems but this is a result of the fact that tetraloops are the most abundant hairpin loop in the data base (16,055 occurrences). Pentaloop hairpins have a slightly higher proportion of bulge loops embedded in their stems (10.4% and 10.2%, for pentaloop and tetraloop hairpin stems, respectively). Beyond hairpin loops of 9, the number of bulge loops embedded in hairpin stems drops off so that for hairpin loops of 10, only 44 occurrences are found in the database; <5% of the hairpins of this size have a bulge loop embedded in their stems. Only 80 occurrences of bulge loops are found one base pair from the hairpin loop in the database. The great majority, almost 80%, of the bulge loop occurrences are found 2–4 bp from the hairpin loop. This percentage would be even higher except for the hairpin tetraloop with a conserved bulge loop (278 occurrences) 7–8 bp from the stem at position 95 in the SSU RNA (*Escherichia coli* numbering).

Finally, we wanted to determine if there was any correlation between the thermodynamics, structure mapping, and arrangement of naturally occurring group II sequences imbedded into hairpin loops. Of the 419 group II bulge loops embedded within a hairpin stem, the bulge loops are evenly divided between whether the bulge loop was embedded in the 5′ or 3′ stem of the hairpin. This is again in concordance with the thermodynamics of insertion of a group II bulge loop into a hairpin stem (Lim et al. 2012). Surprisingly, when the position of the paired nucleotide (proximal or distal to the hairpin loop) was examined, there was no preference for the proximal base being paired. Both the thermodynamics of and structure mapping of bulge loops embedded into hairpins have shown the proximal base to be paired. This divergence may represent the constraints of tertiary structure formation or may be indicative of the structural flexibility imparted by the insertion of a bulge loop into RNA.

### Structure of naturally occurring group II bulge loops in the context of a hairpin stem

RNA FRABASE was used to search for three-dimensional structures with group II bulge loops located between 1 and 8 bp from a hairpin loop of size three to eight (Popenda et al. 2010). A total of 15 examples were identified. The secondary and tertiary structure of the bulge loops are shown in Table 5 (Zimmermann and Singh-Bergmann 1979; Kalurachchi and Nikonowicz 1988; Smith and Nikonowicz 1998; Schmitz and Tinoco 2000; Schlünzen et al. 2001; Fourmy et al. 2002; Ogle et al. 2002; Bashan et al. 2003; Berk et al. 2006; Walden et al. 2006; Tishchenko et al. 2008; Huang et al. 2010; Llano-Sotelo et al. 2010; Stoddard et al. 2010; Klinge et al. 2011; Zhu et al. 2011). All but one of the bulge loop structures displays a structure previously identified (Blose et al. 2007). Nearly half of the determined structures (7 of 15) have the bulge oriented in an extrahelical orientation (panels B, E, G, H, I, J, and N) (Table 5). Four of the structures in Table 5 have the bulge oriented in an intercalated geometry (panels A, K, L, and O). In panels C, F, and M, the bulged base forms a base triple (side-by-side geometry). The last example is shown in panel D; in this instance, the bulged base causes the stem of the hairpin to lose its base-pairing and the bases of the stem are offset. The reason for this offset may be that in this instance, the hairpin loop is a strained triloop. The slippage of the stem base pairs allows the hairpin loop to become more tetraloop like with a GAAA sequence.

**TABLE 5.**
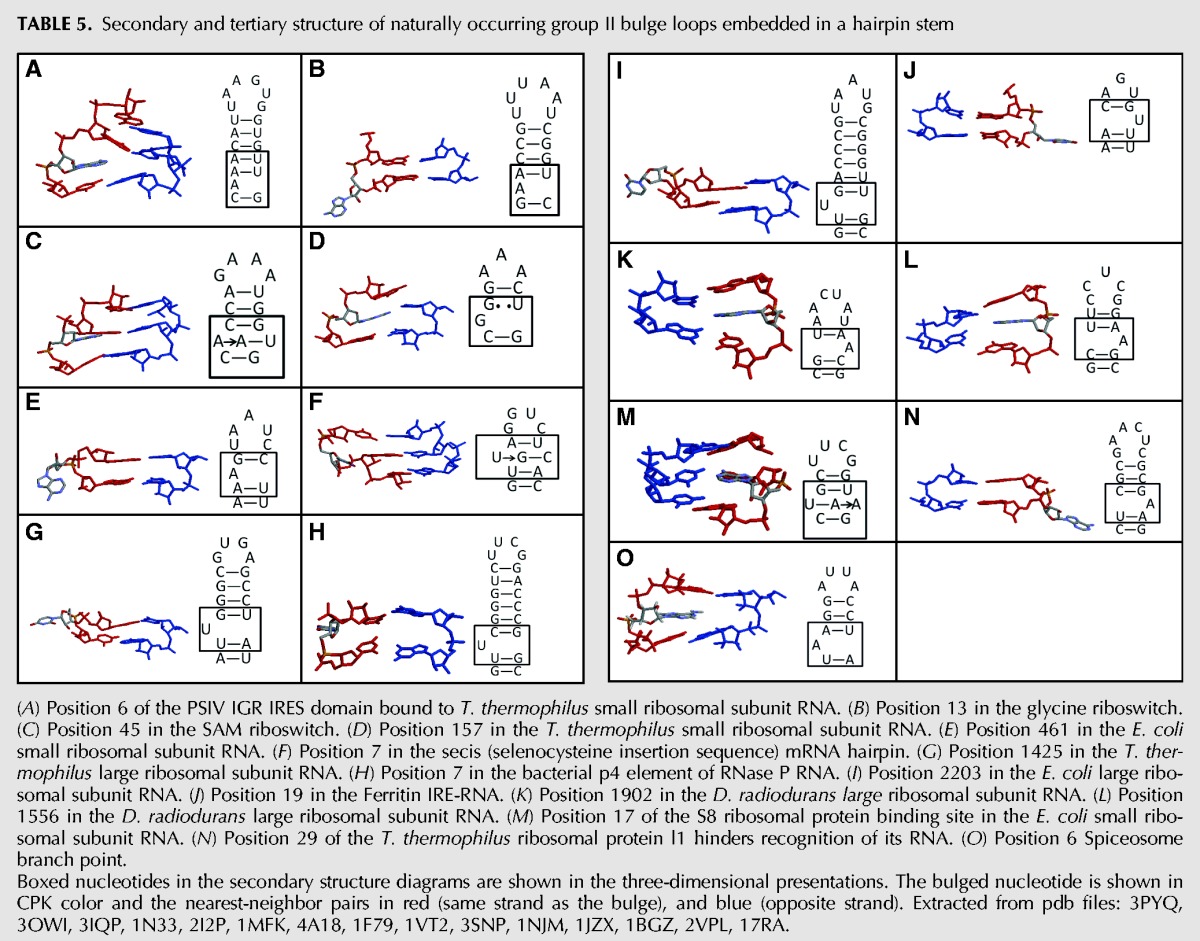
Secondary and tertiary structure of naturally occurring group II bulge loops embedded in a hairpin stem

As observed with the phylogenetic data, the position of the bulged nucleotide is about evenly divided with six of the structures having the bulge in the proximal position (panels E, G, H, I, J, and N) and six having the distal nucleotide as the bulge (panels A, B, D, K, L, and O). For the group II bulge loops with A as the bulge, five of the seven (panels A, B, K, L, and O) have the distal nucleotide unpaired. Interestingly, when the group II bulge is UU, all four examples have the proximal U bulged (panels G, H, I, and J).

Of the 12 (extrahelical and intercalated) naturally occurring hairpin structures in Table 5, eight (panels A, B, E, G, J, K, L, and N) have the bulge loop positioned so that the more stable nearest-neighbor interaction it disrupted by the bulge. Thus, as with the hairpins investigated in this study, the positioning of the bulge does not appear to be influenced by the stability of the nearest-neighbor interaction. Interestingly, of the four examples (panels D, H, I, and O) where the bulge disrupts the less stable nearest-neighbor interaction, a wobble base pair nearest-neighbor interaction is disrupted irrespective of which potential base (distal or proximal) is bulged.

The location and interaction of the extrahelical bulge loops were further examined in the context of the three-dimensional structures. The extrahelical bulge loops are involved in a number of tertiary interactions (data not shown). In four of the seven examples, the bulge interacts with another nucleotide, the bulged nucleotide in panels B, I, and N is stacked on an adenosine residue and the bulge nucleotide in panel G hydrogen bonds with a guanine residue. In one example, the bulge loop in panel J stacks on a histidine residue of the bound protein. In the last two examples the bulges in panels E and H are positioned into a pocket devoid of obvious interactions.

In the structures considered above the geometry of the bugle loop may be influenced by tertiary and crystal packing forces in addition to the local bulge environment. To more closely examine the local influences experienced in solution by the hairpins we investigated by optical and fluorescent melting, we next examined the structure of adenosine bulge loops (not necessarily group II, nor necessarily embedded in a hairpin stem) whose structures were determined by solution NMR analysis. Five examples were identified (Smith and Nikonowicz 1998 [Table 5, panel O]; Thiviyanathan et al. 2000; Newby and Greenbaum 2002; Sashital et al. 2003; Schmitz 2004). In one example (Schmitz 2004), the bulged adenosine forms a base triple with and is stacked on the closing base pairs. In the other examples, the adenosine is intrahelical and also stacked on the closing base pairs. In each of these examples, the bulge leads to a bending of the helix resulting in a nonparallel stacking between the bulged base and its nearest neighbors. It is likely that in the denatured single stranded form, the adenosine is able to stack more favorably with its nearest neighbors resulting in greater nonradiant energy transfer and a decrease in fluorescence. While this represents only a small set of examples, the absence of any extrahelical structures suggests that in the absence of tertiary contacts, adenosine bulges are dynamic but favor stacked conformations.
